# Identification of 11-amino acid peptides that disrupt Notch-mediated processes in *Drosophila*

**DOI:** 10.1186/1423-0127-18-42

**Published:** 2011-06-17

**Authors:** Haiwei Pi, Yi-Chun Huang, I-Chun Chen, Chung-De Lin, Hsiao-Fong Yeh, Li-Mei Pai

**Affiliations:** 1 Department of Biomedical Sciences, School of Medicine, Chang Gung University, 259 Wen-Hwa 1st Road, Kwei-Shan, Tao-Yuan 333, Taiwan; 2 Graduate Institute of Biomedical Sciences, School of Medicine, Chang Gung University, 259 Wen-Hwa 1st Road, Kwei-Shan, Tao-Yuan 333, Taiwan

## Abstract

**Background:**

The conserved Notch signaling pathway regulates cell fate decisions and maintains stem cells in multicellular organisms. Up-regulation of Notch signaling is observed in several types of cancer and is causally involved in proliferation and survival of cancer cells. Thus, it is of great interest to look for anti-Notch reagents for therapeutic purposes. In model animal *Drosophila*, Notch signaling restricts selection of sensory organ precursors (SOPs) during external sensory (ES) organ development. To look for novel genes that can suppress Notch signaling, we performed a gain-of-function modifier screen to look for genes that enhance the phenotype of ectopic ES organs induced by overexpression of *phyllopod*, a gene required for SOP specification.

**Results:**

From the gain-of-function screen, we discovered that overexpression of *polished rice*/*tarsal-less *(*pri/tal) *increases the numbers of ES organs as well as SOPs. *pri/tal *is a polycistronic gene that contains four short open reading frames encoding three 11-amino acid and one 32-amino acid peptides. Ectopic expression of the 11 amino-acid peptides recapitulates the *pri/tal *misexpression phenotype in ectopic ES organ formation. In situ hybridization experiment reveals that *pri*/*tal *mRNA is expressed in the SOPs of the chemosensory organs and the stretch-sensing chordotonal organs.

In *Drosophila *wing development, the Notch signaling pathway mediates the formation of the dorsal-ventral (DV) compartmental boundary and the restriction of the vein width from the primordial veins, the proveins. We also found that *pri*/*tal *mRNA is expressed in the DV boundary and the longitudinal proveins, and overexpression of Pri/Tal peptides disrupts the DV boundary formation and helps to expand the width of the wing vein. Genetic analyses further show that a *Notch *loss-of-function allele strongly enhances these two phenotypes. *Cut *and *E(spl)mβ *are target genes of the Notch pathway in DV boundary formation and vein specification, respectively. We also found that overexpression of Pri/Tal peptides abolishes Cut expression and co-expression of Pri/Tal peptides with *phyl *strongly reduces *E(spl)mβ *expression.

**Conclusions:**

We show for the first time that the overexpression of Pri/Tal 11-amino acid peptides disrupts multiple Notch-mediated processes and reduces Notch target gene expression in *Drosophila*, suggesting that these peptides have novel antagonistic activity to the Notch pathway. Thus, our discovery might provide insights into designing new therapeutic reagents for Notch-related diseases.

## Background

The Notch pathway is an evolutionally conserved signaling system required in a wide range of developmental processes and the maintenance of stem cells [1-3]. Malignancies including T-cell acute lymphoblastic leukemia [4], breast cancer [5], pancreatic cancer [6], lung cancer [7] and ovarian cancer [8] are associated with up-regulation of the Notch signaling activity. Inhibition of Notch signaling pathway has been shown to deplete stem-like cells and suppress the tumor-forming activity in brain tumors [9], and suppress proliferation and induce apoptosis of ovarian and lung cancer cells [7,8].

One excellent model to study the Notch signaling pathway is the development of the fruit fly *Drosophila melanogaster*. During *Drosophila *development, the Notch pathway is involved in developmental processes such as the selection of neural precursors and the specification of wing veins and wing margins [2,10,11]. The *Drosophila *wing veins are formed with a prominent and invariant pattern in adult wings. During larval development, formation of longitudinal vein is initiated by the specification of proveins in the wing imaginal discs. Further restriction of the provein width from eight or nine-cells to two or three-cells requires the activation of the Notch pathway during the pupal stage. In lateral provein cells, the activation of receptor Notch (N) by its ligand Delta expressed in the central region leads to the suppression of vein cell differentiation [12,13]. In *N *loss-of-function mutants, lateral provein cells differentiate into vein fate, causing the widening of wing veins [14].

The Notch pathway is also required to define the dorsal-ventral (DV) compartmental boundary of the wings. Transduction of the Notch pathway at the DV boundary activates downstream targets such as genes encoding the signal molecule Wingless (Wg) and the homeodomain transcription factor Cut [15-17]. When *N*, *wg *or *cut *activity at the DV boundary is disrupted, notched adult wings are detected along the margin.

One classical model to study the role of the Notch pathway in neurogenesis is the development of *Drosophila *sensory organs. Sensory organ development is initiated by basic-helix-loop-helix (bHLH) proneural proteins that are first expressed in neural-competent proneural clusters of cells, in which each cell in the cluster is endowed with the potential to become the sensory organ precursor (SOP) [18,19]. The expression of bHLH proneural proteins in proneural clusters is further restricted to and refined in single cells, the future SOPs, through the Notch pathway [11,20]. In mutants with reduced Notch signaling activity, ectopic SOPs are specified within the proneural clusters or the proneural stripes, leading to the generation of ectopic SOPs and an increase in the ES organ density [21-24].

In order to identify novel genes regulating SOP specification and potentially the Notch pathway, we performed a gain-of-function screen to identify genes that modify the phenotype of ectopic external sensory (ES) organs induced by overexpression of *phyllopod *(*phyl*). The *phyl *gene is a direct downstream target of proneural proteins and is required for SOP specification and ES organ formation [25,26]. Among the six modifiers identified in the screen, we focused on gene *polished rice*/*tarsal-less *(*pri*/*tal*). *pri*/*tal *enhances the *phyl-*overexpression phenotype in the formation of ectopic ES organs. It encodes a polycistronic mRNA that contains four short open reading frams (ORFs) of 36 and 99 base pairs in length (Additional File 1, Figure S1). These four short ORFs express four peptides (Pep1-4). Pep1, Pep2, and Pep3 have only 11 amino acids with Pep1 and Pep2 being identical. Pep4 has 32 amino acids. All four peptides share the core motif of LDPTGQ(T)Y that is present twice in Pep4 [27,28]. These four peptides function redundantly in embryogenesis. *pri*/*tal *regulates the formation of dentical belts and tracheal system in embryos [27,29], and is required for developmental patterning of the legs [28,30]. In this study, we found that overexpression of Pri/Tal peptides causes ectopic ES organ formation, wing vein expansion, and defects in formation of the compartmental boundary of wing imaginal discs. These phenotypes are similar to those seen in *Notch *loss-of-function mutants [14,24]. We further demonstrated that overexpression of Pri/Tal peptides abolishes or helps to reduces the expression of *Cut *and *E(spl)mβ*, Notch target genes in mediating DV boundary formation and suppression of vein specification, respectively. Thus, our gain-of-function screen in model animal *Drosophila *reveals the potential activity of Pri/Tal peptides as peptide antagonists of Notch signaling.

## Methods

### Fly Genetics

All flies were incubated at 25°C unless indicated otherwise. The following flies were used: *UAS-myc-phyl *[25], *UAS-pri*/*tal *(this study), *UAS-pep1*, *UAS-pep3*, and *UAS-pri/tal*^*1-4FS *^[27], *N*^*55e11 *^[23], *UAS-N *[31], *E(spl)mβ-lacZ *[12], *Eq-Gal4 *[25], *dpp-Gal4 *[32], *ato*^*1 *^and *Df(3R)p*^*13 *^[33], *sc*^*10-1*^[34], and *cbl*^*F165 *^[35]. The Pep1-misexpression mitotic clones were generated in *hs-Flp/+; actin > Y+ > Gal4 UAS-GFP/UAS-pep1 *larvae. For the EP gain-of-function screen, crosses were set up with males from 1075 independent EP lines and females of *Eq-Gal4 UAS-phyl/TM6B*. Among them, 273 lines were obtained from the Berkeley *Drosophila *genome projects. The other 802 newly generated lines were provided by Dr. Cheng-Ting Chien.

### In situ RNA hybridization

The same protocal described by Tautz and Pfeifle was used for in situ hybridization [36]. The full-length cDNA (from transcription start +1 to +1532) from EST clone LD11162 was used as the template to generate the *pri*/*tal *anti-sense probe.

### Immunohistology and X-gal staining

For immunohistology, dissected discs or pupal nota were fixed in 4% formaldehyde or 4% freshly prepared paraformaldehyde for 15 minutes. After washing with 1X PBT (phosphate buffered saline with 0.1% TX-100), discs were incubated with anti-Cut (1:1000) or anti-Hnt (1:25) antibodies (Hybridoma Bank) followed by a Cy3-conjugated secondary antibody. For X-gal staining, dissected discs were fixed in 0.3% glutaraldehyde for 10 minutes. After washing in 1XPBS for 3 minutes, the discs were incubated in an X-gal buffer for 30 minutes at 37°C.

### Fluorescence Quantification

The protein levels of Cut were quantified by measuring the immunofluorescence intensity of anti-Cut antibody staining. All samples were imaged in a single plane and images were analyzed using ImageJ software. To determine the mean fluorescence intensity (FI) of ectopically expressed Cut (FI^ectopic^) within the misexpression clones, 15 Cut- and GFP-positive cells that are adjacent or close to the DV boundary were randomly selected for measurement of the Cy3 intensity. In the same disc, the Cy3 intensity of 15 Cut-positive and GFP-negative cells at the DV boundary and 15 Cut- and GFP-negative cells flanking the DV boundary was measured as the mean endogenous (FI^endo^) and background (FI^back^) intensity, respectively. The relative FI was calculated as [(FI^ectopic^- FI^back^)/( FI^endo^- FI^back^)]×100%. For three co-expression clones of Pep1 and N in which the ectopic Cut expression was barely detected, 15 GFP-positive cells flanking the DV boundary were randomly selected for fluorescence measurement.

## Results

### Identifying candidate genes in ES organ development using an EP overexpression screen

**Figure 1:**
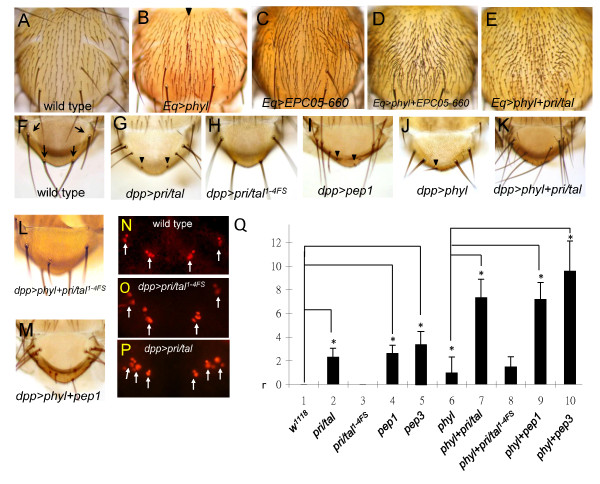
**Forced expression of Pri/Tal peptides induces ectopic ES organ formation**. (A-E) Adult nota. (A) Notum of a wild-type fly. (B) Ectopic expression of *phyl *by *Eq-Gal4 *induces ectopic ES organs, particularly at the midline region (indicated by arrowhead). (C) The ES organ pattern is largely unaffected in notum expressing *EPC05-660 *by *Eq-Gal4*. (D-E) Coexpression of *phyl *and *EPC05-660 *(D) or *phyl *and *pri*/*tal *(E) by *Eq-Gal4 *induces formation of numerous extra ES organs on the notum. (F-M) Adult scutella. (F) Four ES organs (indicated by arrows) located at the wild-type scutellum. (G-I) *dpp-Gal4*-driven expression of *pri*/*tal *(G) or *pep1 *(I) but not frame-shift mutant *pri/tal*^*1-4FS *^(H), induces ectopic ES organs at the scutellum. In some ES organs, the length of the shaft is reduced (indicated by arrowheads) by ectopic expression of *pri*/*tal *or *pep1*. (J) Expression of *phyl *alone by *dpp-Gal4 *generates few ectopic ES organs. The length of the shaft is reduced in some *phyl*-misexpression ES organs (arrowheads). (K and M) Coexpression of *phyl *and *pri*/*tal *(K) or *phyl *and *pep1 *(M) by *dpp-Gal4 *produces many extra ES organs. (L) Coexpression of *phyl *and *pri*/*tal*^*1-4FS *^by *dpp-Gal4 *results in few ectopic ES organs, a phenotype similar to (J). (N-P) In wild-type (N) or *dpp-Gal4 *>*pri*/*tal*^*1-4FS *^(O) pupae at 12-16 hr APF, only four Hnt-positive clusters are observed at the scutella (arrows). In *dpp-Gal4 > pri*/*tal *pupae, more than four Hnt-positive clusters are found. (Q) Quantification of the number of the ectopic scutellar ES organs induced by *dpp-Gal4*. Flies were grown at 18°C for all *dpp-Gal4 *experiments shown in (F) to (Q).

Microchaete, the prominent feature of ES organs in the adult notum, are arranged in regular longitudinal rows. Within a row, each microchaete is evenly spaced and well separated from the others by three to five intervening epidermal cells (Figure 1A). The organized pattern of microchaete makes it an ideal system for use as a genetic modifier screen to identify genes involved in sensory organ development. The E3 ligase adaptor, Phyl, is essential for SOP specification and ES organ formation [25]. Overexpression of *phyl *by *Eq-Gal4 *induces ectopic ES organs on the notum, in particular, in the midline region (arrow in Figure 1B) [25]. A gain-of-function enhancer promoter (EP)-based modifier screen [37] was performed to identify genes that enhance or suppress the *phyl*-overexpression ES organ phenotypes. From 802 newly generated EP lines, six lines significantly modified the *phyl-*overexpression ES organ pattern (Additional File 2, Figure S2). *EPC05-441*, the line that enhanced the *phyl*-overexpression phenotype (Additional File 2, Figure S2D), was located upstream of *charlatan (chn)*, which encodes a zinc-finger transcriptional factor and is required for maximal expression of proneural genes [38]. *EPC05-346*, the line that suppressed the *phyl*-overexpression phenotype, was inserted upstream of *E(spl)-C*, the downstream targets of the Notch pathway that inhibit ES organ formation [39,40]. The identification of *charlatan *and *E(spl)-C *and their involvement in ES organ development validates the effectiveness of this screen. Table 1 lists all six EP lines for their insertion sites and their ES organ phenotypes in expression with or without *phyl*.

**Table 1:** EP screen to look for genes involved in ES organ development.

EP line	*Eq-G4 UAS-phyl*/*EP*	*Eq-Gal4*/*EP*	Gene located downstream of EP insertion
A02-050	ES organ missing	ES organ missing	*escargot*

C05-346	ES organ missing	ES organ missing	*E(spl) complex*

C05-439	ES organ missing	ES organ missing	*CG10588*

C05-441	ES organ density increased	ES organ density increased	*Charlatan*

C05-544	disorganized ES organ pattern	disorganized ES organ pattern	*debra*

C05-660	ES organ density increased	wild-type	*polished rice*/*tarsal-less*

### The overexpression of Pri/Tal peptides induces ES organ formation

This study focused on *EPC05-660 *as it exhibited a strong synergistic interaction with *phyl *in ES organ formation. While the expression of *EPC05-660 *alone by *Eq-Gal4 *induced almost no ectopic ES organs (Figure 1C), coexpression of *UAS-phyl *and *EPC05-660 *induced more ES organs than the expression of *UAS-phyl *alone (Figure 1D). *EPC05-660 *was inserted 191 base pairs upstream of the transcription start site of *pri*/*tal*. To examine whether the ectopic ES organ formation resulted from the expression of *pri/tal*, a *pri/tal *cDNA transgene under the control of *UAS *was generated, which, presumably, produce all four small Pri/Tal peptides. Coexpression of *UAS-pri/tal *and *UAS-phyl *by *Eq-Gal4 *also induced many ectopic ES organs on the notum (Figure 1E), a phenotype similar to the coexpression of *EPC05-660 *and *phyl*.

To quantify the numbers of ectopic ES organs, *dpp-Gal4 *was used to drive *pri*/*tal *and *phyl *expression at the scutellum where only four ES organs are present in wild-type (Figure 1F). The quantification results are shown in Figure 1Q. The transgene *UAS-pri/tal*^*1-4FS*^, which carries frame-shift mutations in the coding sequences of all four small peptides, was used as a control [27]. While the expression of *pri*/*tal *or *phyl *alone induced a few ectopic ES organs (2.3 ± 0.7 and 1 ± 1.3, respectively, Figure 1G and 1J), coexpression of *pri/tal *and *phyl *by *dpp-Gal4 *induced 7.4 ± 1.5 ectopic ES organs (Figure 1K). The expression of *UAS-pri/tal*^*1-4FS *^by *dpp-Gal4 *produced no ectopic ES organs (0 ± 0, Figure 1H) and failed to enhance the *phyl*-overexpression phenotype (1.5 ± 0.8, Figure 1L).

We next investigated whether the expression of single Pri/Tal peptides is sufficient to synergize with *phyl *to induce ES organs. Pep1 or Pep3, when coexpressed with Phyl, induced as many as 7.2 ± 1.4 ectopic ES organs with Pep1 and 9.6 ± 2.5 organs with Pep3 (Figure 1M and data not shown). Only a few ectopic ES organs were detected in flies expressing either Pep1 (2.6 ± 0.7) or Pep3 (3.3 ± 1.3) alone (Figure 1I and data not shown). While the analysis using *dpp-Gal4 *suggested a synergistic interaction between single Pri/Tal peptides and Phyl, it also indicated that Pri/Tal peptides alone are sufficient to promote ES organ formation in the absence of Phyl coexpression.

Each ES organ is composed of four cells (neuron, sheath cell, shaft cell and socket cell) which are derived from asymmetric cell division of a single SOP. The ability of Pep1 and Pep3 to induce ectopic ES organs suggests that Pri/Tal peptides are sufficient to promote formation of the SOPs. By using antibody recognizing Hindsight (Hnt) protein [41], a marker for SOPs and the SOP progenies, four Hnt-positive clusters were observed at the wild-type pupal scutellum at 12-16 hr APF (after puparium formation) (arrows in Figure 1N). These four clusters of cells correspond to the SOP progenies of the four endogenous scutellar ES organs. In pupae expressing *pri*/*tal *through *dpp-Gal4*, more than four Hnt-positive clusters were observed (arrows in Figure 1P), indicating the formation of ectopic SOPs. No ectopic Hnt-positive cluster was found at the scutellum of *dpp-Gal4 *>*UAS-pri/tal*^*1-4FS *^pupae (arrows in Figure 1O). Thus, ectopic SOP formation can be induced by overexpression of Pri/Tal peptides.

### Expression patterns of *pri*/*tal *in imaginal discs

**Figure 2:**
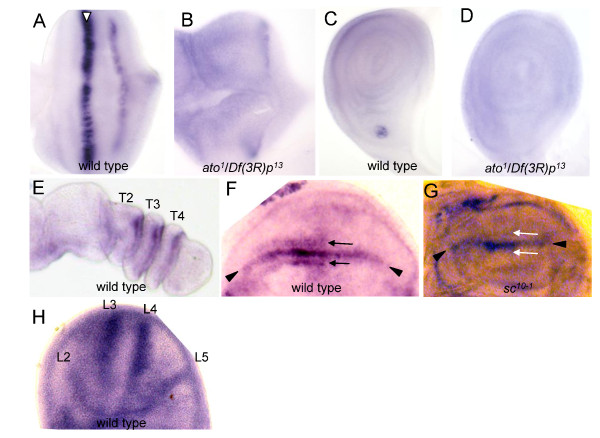
***pri*/*tal *is expressed dynamically in imaginal discs**. and C) *pri*/*tal *mRNA is detected in the R8 preclusters in eye discs (arrowhead in A) and in the chordotonal organ precursors in leg discs (C). (B and D) In *ato*^*1*^/*Df(3R)p*^*13 *^mutants, *pri*/*tal *mRNA levels in the R8 clusters (B) and chordotonal organ precursors (D) are eliminated. (E) *pri*/*tal *mRNA is detected in the tarsal leg joints at 4-8 hr APF. T2-T4 mark the second to fourth tarsal segments. (F) *pri/tal *is expressed at the DV boundary (arrowheads) and in the precursors of anterior chemosensory organs (arrows). (G) No *pri*/*tal *mRNA is detected in the anterior chemosensory organ precursors in *ac sc *null mutants (*sc*^*10-1*^) (white arrows). (H) Wing disc of 0-4 hr APF. *pri*/*tal *mRNA is detected in the proveins of longitudinal veins L2-L5.

The adult structures of *Drosophila *are developed from imaginal discs, small sacs of epithelium present in larvae and early pupae. To further characterize the roles of Pri/Tal peptides in development, *pri*/*tal *mRNA patterns were examined in imaginal discs by in situ hybridization with probes specific to *pri*/*tal*. In eye discs, *pri*/*tal *mRNA was detected in the preclusters for presumptive R8 photoreceptors and in a stripe of cells in the posterior region of eye discs whose fate was not determined (Figure 2A). In leg discs, a cluster of precursors to develop into stretch-sensing chordotonal organs expressed *pri*/*tal *(Figure 2C). These patterns suggest that *pri*/*tal *expression is under the control of the proneural protein Atonal (Ato) [33,42]. The expression of *pri*/*tal *in R8 preclusters and chordotonal organ precursors were consistently eliminated in loss-of-function *ato*^*1*^*/Df(3R)p*^*13 *^mutants (Figure 2B and 2D), indicating that Ato is a key upstream regulator for *pri*/*tal *expression in these two developing tissues.

In larval leg discs, *pri*/*tal *is expressed in the tarsal region, and is required for specification of tarsal segments [28]. At 4-8 hr APF, *pri*/*tal *mRNA was concentrated at the presumptive joints between tarsal segments (Figure 2E). In wing discs of third instar larvae, *pri*/*tal *mRNA was present at the DV compartmental boundary (arrowheads in Figure 2F) and in two stripes of cells straddling the anterior part of the DV boundary (arrows). The latter expression pattern suggests that *pri*/*tal *is expressed in the precursors for chemosensory organs. The formation of chemosensory organs along the anterior wing margin depends on the activities of the proneural genes *ac *and *sc*. In the *sc*^*10-1 *^mutant that abolishes both *ac *and *sc *activities, *pri*/*tal *mRNA expression in these two anterior stripes was abolished (white arrows in Figure 2G). From the late third instar larval to the early pupal stages, *pri*/*tal *mRNA was also detected in the provein cells that develop into longitudinal wing veins L2-L5 (Figure 2H and data not shown).

### Overexpression of Pri/Tai peptides promotes vein cell fate

**Figure 3:**
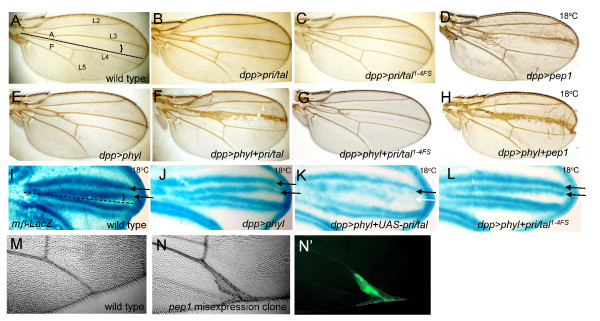
**Wing vein expansion induced by overexpression of Pri/Tal peptides**. (A-H) Adult wings. (A) Wild-type wing. The AP boundary is indicated by the dotted line, and the *dpp-Gal4 *expression region is marked by the bracket. (B-H) Strong L3 vein expansion and formation of a large number of ES organs on the L3 vein are observed in flies co-expressing *phyl *and *pri*/*tal *(F), or in flies co-expressing *phyl *and *pep1 *(H). Expression of *pep1 *also induces mild vein expansion at the tip of L3 vein (D). (I-L) X-gal staining of the wing discs dissected from *E(spl)mβ-lacZ *pupae at 20-24 hr APF. The black arrows indicate the L3 vein boundaries expressing normal levels of *E(spl)mβ-lacZ*. White arrow in (K) points to the reduced *E(spl)mβ-lacZ *expression at the posterior boundary of the L3 vein. (M) Wild-type wing. (N and N') Wing vein expansion is also observed in the *pep1*-misexpressioin clone. The misexpression clone is identified by the presence of GFP (N') and the clone boundary is marked by black dash lines in (N).

The *dpp-Gal4 *driver is expressed in a band of anterior cells adjacent to the anterior-posterior (AP) boundary of the wing discs (bracket in Figure 3A) [43]. This expression pattern overlaps the posterior region of the L3 provein. While expression of *phyl *alone by *dpp-Gal4 *had no effect on the morphology of the L3 vein (Figure 3E), coexpression of *pri/tal *and *phyl *caused strong L3 vein expansion (Figure 3F) that was accompanied by ectopic ES organ formation. Expansion of the L3 vein and induction of a large number of ES organs require the coexpression of Pri/Tal peptides and Phyl. This is because these phenotypes were not found in the coexpression of *pri/tal*^*1-4FS *^and *phyl *(Figure 3G) or in the flies expressing *pri*/*tal *(Figure 3B). The expression of Pep1 or Pep3 alone induced mild but detectable L3 vein expansion at the distal tip even at 18°C, a temperature used to reduce Gal4 activity [44] to avoid lethality by *dpp-Gal4*-driven overexpression (Figure 3D and data not shown).

During wing vein development, Notch signaling, that refines provein to vein regions, is activated at both the anterior and posterior boundaries of proveins. This is seen by the expression of the Notch pathway target reporter *E(spl)mβ-lacZ *(arrows in Figure 3I). The expression of *phyl *or *pri*/*tal *alone by *dpp-Gal4 *did not alter this *E(spl)mβ-lacZ *pattern (Figure 3J and data not shown). In the coexpression of *pri/tal *and *phyl*, however, the *lacZ *expression at the posterior L3 provein boundary was severely disrupted (white arrow for posterior boundary in Figure 3K). Similar results were observed in the coexpression of Phyl and Pep1 (data not shown). In the control wing discs in which *phyl *was coexpressed with *pri*/*tal*^*1-4FS*^, the *lacZ *patterns and levels remained indistinguishable from the wild-type discs (Figure 3L).

To test whether expression of the Pri/Tal peptides also affects formation of the other veins, ectopic Pep1-expressing GFP-marked clones under the *actin *promoter were generated (see Methods). In ten clones that overlapped wing veins, as visualized by GFP expression (Figure 3N'), three displayed vein expansion phenotype (Figure 3N). In many more clones that did not overlap wing veins, no ectopic vein was observed, suggesting that Pep1 alone is sufficient to promote vein fate from the provein cells.

### Overexpression of Pri/Tal peptides disrupts DV boundary formation

**Figure 4:**
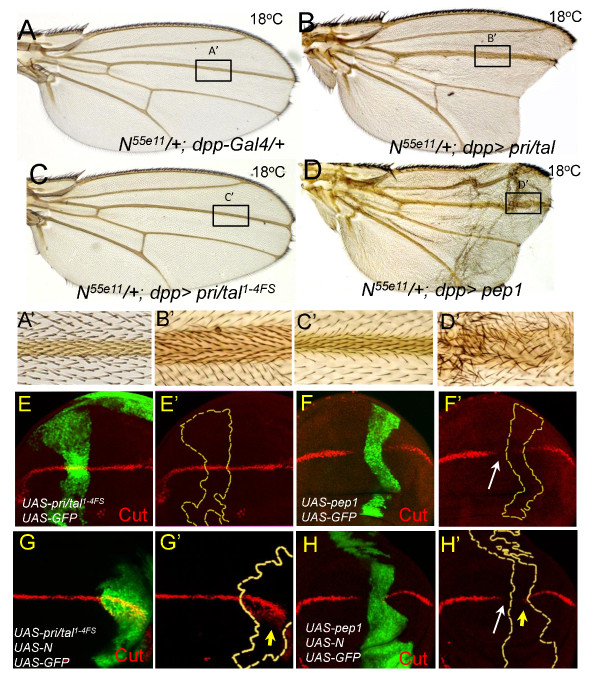
**Overexpression of Pri/Tal peptides disrupts the DV boundary formation of the wing discs**. (A-D) Expression of *pri*/*tal *(B) or *pep1 *(D) by *dpp-Gal4 *causes L3 vein expansion and notched wing in *N*^*55e11*^/+ background. (A'-D') Higher magnification of the insets in (A) to (D). (E-H) In all figures, Cut expression is shown in red and the misexpression clones are identified by the presence of GFP (green) and marked by the yellow dash lines in (E'-H'). (E) Cut expression at the DV boundary is not affected in clones of *pri/tal*^*1-4FS*^. (F) In *pep1 *clones, Cut levels within or adjacent to the boundaries of the clones are strongly reduced. White arrow indicates the non-autonomous suppression of Cut expression by Pep1. (G and H) Expression of *pep1 *(H), but not *pri/tal*^*1-4FS *^(G), suppress ectopic Cut expression (indicated by yellow arrows) activated by the receptor, N.

A recent study of *phyl *in the developing *Drosophila *eye shows that Phyl, by facilitating Notch trafficking to lysosome, promotes Notch protein degradation [45]. Thus, the disruption of *E(spl)mβ-lacZ *patterns by the coexpression of *pri*/*tal *and *phyl *suggested further examination of the genetic interaction between *pri*/*tal *and *N*. In the sensitized *N*^*55e11*^*/+ *animal in which one copy of the *N *gene is inactivated, L3 veins were mildly expanded (compare Figure 4A to 3A). Expression of *UAS-pri*/*tal *by *dpp-Gal4 *in *N*^*55e11*^*/+ *strongly enhanced the expansion of L3 veins with complete penetrance (n = 12) (Figure 4B and 4B'). The control *UAS-pri/tal*^*1-4FS *^did not modify the vein of *N*^*55e11*^*/+ *(Figure 4C, 4C'). Expression of Pep1 by *dpp-Gal4 *in *N*^*55e11*^*/+ *also enhanced vein expansion, particularly at the distal region of L3 (Figure 4D and 4D').

As shown in Figure 2F, *pri*/*tal *was also expressed at the DV boundary of the wing discs, the site of the future wing margin. Expression of *pri*/*tal *or Pep1, but not *pri/tal*^*1-4FS*^, strongly enhanced the wing-notching phenotype of *N*^*55e11*^*/+ *wing (Figure 4A-D). The homeodomain protein Cut, required for the formation of the wing margin [17], is expressed at the DV boundary in response to activation of the Notch pathway. Thus, overexpression clones of Pep1 were generated to examine whether Cut expression was affected by Pep1. In all Pep1 clones overlapping the DV boundary, Cut expression was strongly inhibited (100%, N = 11) (Figure 4F and 4F'). Similar results were also found in overexpression clones of *pri*/*tal *(data not shown). In contrast, no reduction of Cut levels was observed in clones expressing *pri*/*tal*^*1-4FS *^(100%, N = 20) (Figure 4E and 4E'). In addition to suppression of Cut levels by the Pep1 within the clones, we found that Cut level was also significantly reduced in non-clonal cells near the clone (white arrow in Figure 4F'). By analyzing Cut levels in twenty-two non-clonal areas that are flanking the clones, we found that Cut expression was strongly suppressed in thirteen of them (59%): ten showed Cut reduction within ten cells from the GFP-positive clones, and three showed Cut reduction in cells that were as far as 10 to 12 cells away from the clone. Thus, Pri/Tal peptides can repress Cut expression at the DV boundary both autonomously and non-autonomously.

Pep1 was further investigated to determine if it could indeed suppress Cut expression induced by the Notch pathway. Overexpression of receptor N together with the non-functional *pri*/*tal*^*1-4FS *^autonomously induced ectopic Cut expression in cells adjacent to the DV boundary (yellow arrow in Figure 4G'), similar to that of misexpression of N alone [46]. However, both ectopic and endogenous Cut expressions in N overexpression clones were repressed by the coexpressed Pep1 (Figure 4H and H'). By quantifying the intensity of the anti-Cut antibody staining (see Methods), we found that the average protein level of ectopically expressed Cut induced by the coexpression of *N *and *pri*/*tal*^*1-4FS *^was 85% of the endogenous Cut at the DV boundary (N = 10). In the coexpression clones of N and Pep1, the average protein level of ectopically expressed Cut was reduced to 23% of the endogenous level (N = 11). At times, it was below 5% (N = 3), which meant that it was barely detectable by immunofluorescent staining (yellow arrow within the clone in Figure 4H'). In the coexpression clones of N and Pep1, the non-autonomous suppression of Cut expression at the DV boundary was also observed in eight out of the seventeen non-clonal areas that flanking the GFP-positive clones (47%) (white arrow in Figure 4H'). As the co-expression result shown in Figure 4H, the Pri/Tal peptide is able to repress Cut expression induced by the activation of the Notch pathway.

## Discussions

The Notch pathway is a highly conserved signaling system that regulates cell proliferation, differentiation and death, and aberrant activation of Notch signaling is associated with several types of tumor [47]. Although γ-secretase inhibitors have been shown to successfully suppress tumor growth in several types of Notch-related cancers [48-51], the cytotoxicity in the GI tract complicates its potential in cancer therapy [52]. Thus, it is important to look for more anti-Notch reagents for therapeutic purposes.

During *Drosophila *development, the Notch pathway is involved in the developmental processes such as selection of neural precursors and specification of wing veins and wing margins [2,10,11]. Overexpression of Pri/Tal peptides leads to phenotypes such as the formation of ectopic SOPs, expansion of the wing veins and disruption of the DV boundary. These are reminiscent of the *N *loss-of-function phenotypes. Overexpression of *pri*/*tal *or Pep1 eliminates or helps to reduce the expression of Notch target genes *Cut *and *E(spl)mβ*. Furthermore, expression of Pep1 represses Cut expression induced by the activation of the Notch pathway. Pri/Tal peptides mediate F-actin assembly in the embryonic tracheal development [27]. The effect of Pri/Tal peptides on cytoskeletal dynamics and how it might contribute to the suppression of the Notch-mediated expression of Cut and *E(spl)mβ *needs further study.

During wing vein development, EGFR and Notch signaling pathways act antagonistically in specifying longitudinal veins [53]. The E3 ligase encoded by *cbl *negatively regulates EGFR signaling by downregulating the level of active EGF receptors [54]. Although *pri*/*tal *acts synergistically with *N*^*55e11 *^to cause wing vein expansion, vein width was not further modified when one copy of *cbl *null allele (*cbl*^*F165*^) was introduced into *dpp-Gal4 *>*UAS-pri/tal *flies (Additional File 3, Figure S3). Thus, the genetic interaction results suggest a more specific effect of *pri*/*tal *to the Notch signaling than to the EGFR pathway in vein fate specification.

A previous report showed that Pri/Tal peptides could function non-autonomously to regulate formation of the larval ventral denticle and adult tarsal segment [27,30]. In developing legs, analyses of *tal *mutant clones show that Tal/Pri-dependent signal is local with a range of 2-3 cells [30]. Our analysis found that Pri/Tal peptides, when overexpressed, can function as far as 12 cells away from the clones to suppress Cut expression at the DV boundary. The difference in the range of non-autonomy might be due to the different expression levels. Alternatively, the extracellular distribution of Pri/Tal peptides might be differentially regulated in different developmental processes, or different peptide concentrations are required for eliciting distinct signaling responses, such as development of leg tarsal segments and suppression of Cut expression at the DV boundary. We also found that the reduction of Cut levels in cells within the clones is stronger than that in cells outside the clone and the severity of the reduction is conversely correlated with the distance from the clones. Therefore, the non-autonomy of Pri/Tal peptides is similar to that of morphogenes, raising the possibility that these extremely small peptides might pass through membranes to exert their non-autonomous effects on neighboring cells.

In the coexpression clones of Pep1 and N, Pep1 also non-autonomously suppressed Cut expression at the developing wing margin in 47% of the non-clonal areas flanking the clones. The non-autonomous suppression effect can be as far as 9 cells from the clones (data not shown). Thus, the presence of N appears not to significantly interfere with the non-autonomous effect of Pep1 to suppress Cut expression.

Although we have shown that overexpression of Pri/Tal peptides, in combination with *phyl*-coexpression or in *N*^*55e11*^*/+*heterozygous background, strongly inhibit Notch-mediated developmental processes in SOP selection and wing development, expression of Pri/Tal peptides alone in wild-type flies only have mild effects on these processes. Thus, decoding the molecular mechanisms of Pri/Tal peptides to the Notch pathway is critically important in the future in order to improve its efficiency as potential anti-Notch reagent.

## Conclusion

By using *Drosophila *development as a model system, we identify and demonstrate for the first time that overexpression of Pri/Tal 11-amino acid peptides promotes SOP and wing vein specification, and suppresses DV boundary formation of the developing wing. These are reminiscent of Notch loss-of-function phenotypes. We also found that overexpression of Pri/Tal peptides abolishes or help to reduce the expression of Notch target genes, suggesting that these small peptides have novel activity to negatively modulate the Notch signaling pathway. Thus, our discovery might provide insights into designing new therapeutic reagents for treating Notch-related malignancies and diseases.

## Competing interests

The authors declare that they have no competing interests.

## Authors' contributions

HP designed the experiments, performed the genetic screen and wrote the manuscript. YCH did the rest of the genetic experiments and help to write the manuscript. ICC did the in-situ hybridization experiment. CDL and HFY helped the genetic experiment and analyzed the data. LMP participated in the design of the study and revised the manuscript. All authors read and approved the final manuscript.

## Supplementary Material

Additional file 1 **Figure S1. Schematic representation of gene organization and peptide products of *pri*/*tal***. The top panel shows the genomic structure of *pri*/*tal*, which contains a single exon (the thick blue bar). The second panel shows the *pri/tal *mRNA (the thin blue bar). Four small ORFs (ORF1-ORF4) located within the *pri/tal *genomic region and the polycistronic mRNA are shown in green. The peptide sequences encoded by these four ORFs are shown as one-letter amino acid abbreviations. The core sequence (LDPTGXY) shared by Pep1 to Pep4 is highlighted in red. Click here for file

Additional file 2 **Figure S2. Strong gain or loss of notal ES organs are observed in flies co-expressing *phyl *and one of the six candidate EP lines by *Eq-Gal4***. Click here for file

Additional file 3 **Figure S3. Overexpression of *pri*/*tal *by *dpp-Gal4 *does not induce L3 vein expansion or ectopic vein in *cbl*^*F165*^/+ background**. Click here for file
