# Supplementary figures and images for: Identification of 11-amino acid peptides that disrupt Notch-mediated processes in Drosophila
Source: J Biomed Sci. 2011 Jun 17;18(1):42. doi: 10.1186/1423-0127-18-42 (PMC3136413; doi:10.1186/1423-0127-18-42)

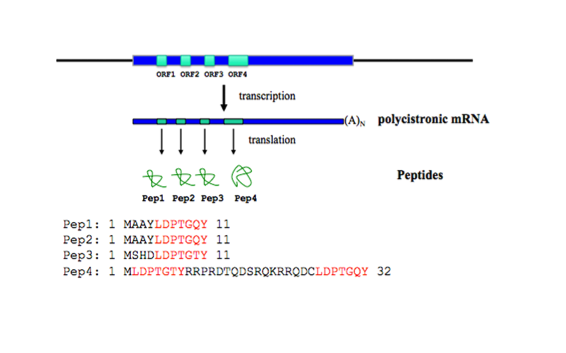

Supplement: Additional file 1 — Figure S1. Schematic representation of gene organization and peptide products of pri/tal. The top panel shows the genomic structure of pri/tal, which contains a single exon (the thick blue bar). The second panel shows the pri/tal mRNA (the thin blue bar). Four small ORFs (ORF1-ORF4) located within the pri/tal genomic region and the polycistronic mRNA are shown in green. The peptide sequences encoded by these four ORFs are shown as one-letter amino acid abbreviations. The core sequence (LDPTGXY) shared by Pep1 to Pep4 is highlighted in red. [file 1423-0127-18-42-S1.TIFF]

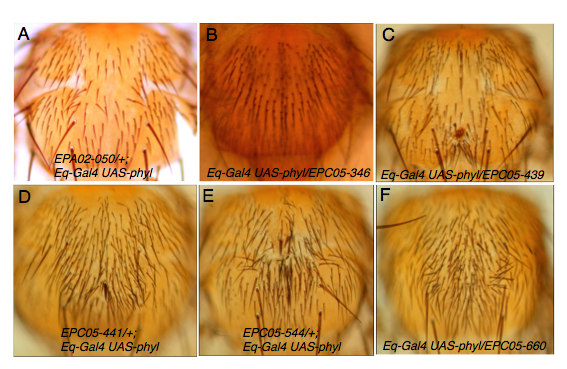

Supplement: Additional file 2 — Figure S2. Strong gain or loss of notal ES organs are observed in flies co-expressing phyl and one of the six candidate EP lines by Eq-Gal4. [file 1423-0127-18-42-S2.TIFF]

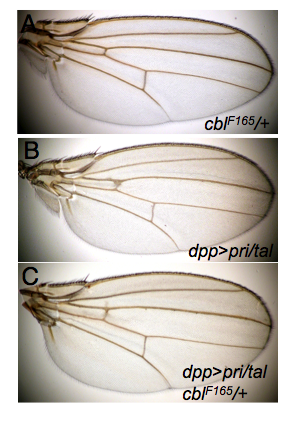

Supplement: Additional file 3 — Figure S3. Overexpression of pri/tal by dpp-Gal4 does not induce L3 vein expansion or ectopic vein in cblF165/+ background. [file 1423-0127-18-42-S3.TIFF]
